# School Drug Testing: A Critical Review of the Literature

**DOI:** 10.1100/tsw.2010.31

**Published:** 2010-03-05

**Authors:** Daniel T.L. Shek

**Affiliations:** ^1^ Department of Applied Social Sciences, Hong Kong Polytechnic University, Hong Kong; ^2^ Department of Sociology, East China Normal University, Shanghai, China; ^3^ Public Policy Research Institute, The Hong Kong Polytechnic University, Hong Kong; ^4^ Kiang Wu Nursing College of Macau, Macao

**Keywords:** drug testing, adolescent substance abuse, abuse detection, adolescents

## Abstract

This paper explores the question of whether school drug testing is an effective solution to tackle adolescent substance abuse problems. Research studies in major academic databases and Internet websites are reviewed. Several observations are highlighted from the review: (1) there are few research studies in this area, particularly in different Chinese contexts; (2) the quality of the existing studies was generally low; and (3) research findings supporting the effectiveness of school drug testing were mixed. Methodological issues underlying quantitative and qualitative evaluation studies of the effectiveness of school drug testing are also discussed.

### INTRODUCTION

A survey of the websites of several international organizations (e.g., Office on Drugs and Crime of the United Nations, International Narcotics Control Board, National Institute of Drug Abuse in the United States, and European Monitoring Center for Drugs and Drug Addiction) shows that illicit drug use is a thorny global problem to be resolved. Probably because of the influence of the popular culture and youth subculture, substance abuse among young people has also become an acute global problem[1,2]. With reference to the findings reported in some of the major databases on adolescent development, such as Monitoring the Future, Youth Risk Behavior Surveillance (YRBS), and National Household Survey on Drug Abuse (NHSDA), adolescent substance abuse is a concern for policy makers and health professionals[3]. From the results of the 2008 National Survey on Drug Use and Health, it was found that 9.3% of youths aged 12–17 were current illicit drug users[4].

To tackle the gradual worsening of adolescent drug abuse, school drug testing has been adopted in some Western countries in order to cope with the problem. In 1995, the U.S. Supreme Court approved drug testing for student athletes in public high schools. In 2002, the U.S. Supreme Court broadened the ruling to include all students taking part in competitions against students at other schools in after-school activities[5]. Ever since its inception, there has been much debate on the necessity and value of student drug testing, particularly its effectiveness. Roche et al.[6] reviewed the theories, assumptions, and limitations of the underlying rationales for school drug testing. They also reviewed some of the studies in the field and concluded that the quality of the studies was generally low. Although the study of Roche et al.[6] is a pioneering attempt to review some of the studies in the field, there are three limitations. First, the studies under review were not exhaustive, as some of the studies reported in academic journals and the Internet were not included. Second, although the quality of the studies under review was discussed in the paper, the details (e.g., problems of the design, methodology and data analyses, biased conclusions, etc.) were not included. Third, findings that support the effectiveness of school drug testing and those that oppose it were not separately reported. Against this background, the present study attempted to review the literature on the effectiveness of school drug testing. Findings from the literature that support it and those that criticize it are individually presented. Besides, quality of the studies is evaluated in detail. Finally, methodological issues intrinsic to quantitative and qualitative studies of the effectiveness of school drug testing are also discussed.

### METHODS

#### Search Strategy

The primary aim of this systematic review was to explore the effectiveness of the contentious issue, school drug testing. Searches were undertaken within the major academic databases: PsycINFO, Social Work Abstracts, Medline, CINAHL, and Sociological Abstracts, using multiple keywords: *random drug test* or *drug testing* or *school drug testing* or *drug detection*. In addition, empirical studies reported in the websites on the Internet were also reviewed using the above terms. The studies under review in the current study are outlined in Appendix 1. Some of the authoritative websites on school drug testing are presented in Table 1.

**TABLE 1**
**Internet Websites on School Drug Testing**

| Resource | Website |
|---|---|
| American Civil Liberties Union - A Test You Can't Study For: Special Web Feature on Student Drug Testing | http://www.aclu.org/drugpolicy/testing/10845res20021021.html |
| Drug Policy Alliance Network | http://www.drugpolicy.org/law/drugtesting/students/ |
| Monitoring the Future | http://www.monitoringthefuture.org |
| Office of Safe and Drug-Free Schools (OSDFS), U.S. Department of Education | http://www.ed.gov/about/offices/list/osdfs/index.html |
| Prevention Resources and Information on Drug Education (PRIDE) | http://www.prideprevention.org/ |
| Student Drug Testing Coalition | http://www.studentdrugtesting.org/ |
| Students for Sensible Drug Policy (SSDP) | http://www.DAREgeneration.com |
| The Association for Addiction Professionals | http://www.naadac.org/ |

### RESULTS

Several observations can be highlighted from the review. First, not many studies have been conducted to examine the effectiveness of school drug testing since its introduction. With particular reference to the Chinese culture, no study has been conducted in different Chinese contexts. Second, most of the empirical studies were cross-sectional in nature (e.g., surveys and qualitative interviews) and not many experimental studies have been conducted. Third, while there are findings from studies that are in support of school drug testing (Table 2), there are also some that do not (Table 3). Fourth, as shown in Table 4, quality of the existing studies was generally not high; therefore, doubt is cast on their conclusions on the effectiveness of drug test. Added to this, there were few well-designed quantitative studies and well-crafted qualitative evaluation studies in the field.

### DISCUSSION

Despite the heightened public concern for school drug testing and its controversial nature, empirical studies that examine the effectiveness of drug testing in the school context are, surprisingly, limited in number. From the perspective of evidence-based practice, research studies play an indispensable role in clarifying the effectiveness of school drug testing and providing support for the policy. As Chinese people constitute roughly one-fifth of the world's population, the absence of school drug testing research is definitely undesirable, particularly in view of the fact that mandatory drug testing is legally acceptable in mainland China. Furthermore, research on school drug testing is indispensable when voluntary school drug testing was implemented in the Tai Po district of Hong Kong on a trial basis in 2009/10 school year.

The present review shows that there are mixed findings on the effectiveness of school drug testing. It is noteworthy that while there are findings that indicate that drug testing had no positive effect, there are findings that support the effectiveness of school drug testing. This picture is also clearly shown in the study of Goldberg et al.[7], which is one of the few prospective trials in the field. As pointed out by Goldberg et al.[7], "although these findings may differ in other schools or regions of the United States, this study *lends credence to some DAT deterrent effects*, especially for past year use for drugs, at two time points, and for drugs and alcohol at two time points. However, because *some substance abuse mediators appeared to worsen and past month substance use never changed*, more research should be performed to assess the policy of drug and alcohol testing's overall effects" (p. 428, italics added). Similarly, Knight and Levy[8], in an editorial of the *Journal of Adolescent Health*[8], pointed out that "although we might hope that the present study by Goldberg would help to end the national debate, this hope is unlikely to be realized on the basis of this report, which includes ample 'evidence' to fuel the fire on both sides" (p. 419).

As far as the quality of the studies under review is concerned, the review shows that the quality of the existing studies was not high. In addition, it is noteworthy that the findings in the studies under review cannot give a definitive answer to the question of whether school drug testing is effective. For example, although a large sample size was used in the study of Yamaguchi et al.[9], the major limitation was its cross-sectional design because "one cannot make definitive causal interpretations regarding effects of drug testing; only a panel design in a randomized or natural experiment can do so. Perhaps schools that instituted drug testing initially had higher use, and drug testing reduced those levels to levels similar to those at other schools" (p. 164). With the aim to explore the association between student drug use and drug-testing policies in schools, Yamaguchi et al.[9] concluded that "while lack of evidence for the effectiveness of drug testing is not definitive, results suggest that drug testing in schools may not provide a panacea for reducing student drug use that some (including some on the Supreme Court) had hoped" (p. 164). However, probably because of the large sample involved, this study has commonly been taken as evidence against school drug testing.

Obviously, the sustainability of school drug testing depends principally on the amount and quality of research evidence supporting its value and effectiveness. There are two lines of evaluation research that should be done in the future. To begin with, quantitative research utilizing experimental designs should be conducted. However, there are at least six issues that should be addressed in experimental studies. First, selection bias (pregroup differences) may confound the results. Studies utilizing pre-experimental designs, such as the one conducted by Yamaguchi et al.[9], are particularly vulnerable to this threat. Second, it is noteworthy that a drug testing scheme will heighten other schools' sensitivity to drug prevention, which may minimize the treatment effect in the experimental groups. Third, it is possible that

## TABLE 2
# A Summary of Findings that Support School Drug Testing

| Study | Study Design | Setting | Sample | Intervention | Outcomes assessed | Findings |
|---|---|---|---|---|---|---|
| Coombs and Ryan (1990) | Cross-sectional survey and in-depth interview | 21 intercollegiate teams, USA | 624 intercollegiate athletes | Mandatory drug testing program | • Identification of student drug users<br>• Prevention of continued drug use | *Drug testing proved to be effective*:<br>• Identification of drug users<br>• Prevention of continued drug use<br>• Reduction of drug use found in most of drug-using athletes |
| Coombs and Coombs (1991) | Cross-sectional survey and in-depth interview | 21 intercollegiate teams, USA | 500 intercollegiate athletes | Mandatory drug testing program | Assessment of students' morale and psychological well-being | Most students were not affected by drug testing.<br>*Positive benefits*:<br>• Promote awareness of negative drug effects<br>• Offer socially acceptable way to resist drug use<br>• Enhance athletic and academic performance |
| DuPont, Campbell, and Mazza (2002) | Cross-sectional survey | Nine high schools, USA | School administrators, counselors, athletic directors, drug-prevention coordinators | Student drug testing | • Students' reported drug use<br>• Disciplinary problems | Decreased students' illicit drug use<br>*Reduced disciplinary problems*:<br>• Lowered detention rate for disruptive behavior<br>• Reduced student arrests |
| McKinney (2002) | Cross-sectional survey | 83 high schools, Indiana, USA | 83 high school principals | Mandatory random drug testing in 1999–2000 | *Compare two academic years: 1999–2000 (when drug testing policies were in effect) to 2000–2001 (when random drug testing policies were suspended):*<br>• Students' reported illicit drug use<br>• Students' alcohol use<br>• Students' suspension or expulsion due to drug or alcohol use | *After the suspension of random drug testing program*:<br>• Increase in illicit drug use<br>• Increase in alcohol use<br>• Statistically significant increase in students' suspension or expulsion |
| McKinney (2003) | Cross-sectional survey McKinney's (2002) follow-up | 59 high schools, Indiana, USA | 59 high school principals | Mandatory, random drug testing program | Students' reported drug and alcohol use when drug testing reimplemented | • Drug testing discouraged students' drug and alcohol use<br>• Decreased students' drug and alcohol use |
| McKinney (2005) | Cross-sectional survey McKinney's (2003) follow-up | 56 high schools, Indiana, USA | 56 high school principals | Mandatory, random, suspicion-less drug testing program | • Students' reported illicit drug use<br>• Students' participation in athletic program<br>• Students' academic performance | • Reduction in students' drug use<br>• Rise in student participation in athletic program<br>*Schools with drug testing*:<br>• Above average in state graduation test<br>• Graduation rate higher than state average |
| Mason (2003) | Cross-sectional survey | High schools, USA | 620 high school students | Drug testing program | • Students' attitude toward drug testing | • Neutral attitude on drug testing in most students<br>• More positive attitude found in younger students<br>• Drug testing less accepting in students with stronger prodrug attitude |
| Goldberg, Elliot, MacKinnon, Moe, Kuehl, Nohre, and Lockwood (2003) | Longitudinal survey 1999–2000 | Two high schools, Oregon, USA | Athletes vs. nonathletes:<br>T1: 276 vs. 507<br>T2: 159 vs. 338 | Mandatory drug testing program for student athletes | • Students' past 30-day drug use<br>• Students' attitude and beliefs on drug testing | • Reduction in the number of student athletes to use drug in the past 30 days<br>• Positive attitude toward drug testing in intervention and control groups |
| Evans, Reader, Liss, Wiens, and Roy (2006) | Cross-sectional survey conducted before drug testing implementation | Two rural high schools, North Florida, USA | 1,011 students from 9th to 11th grade | Random suspicion-less drug testing program | • Students' perceived fairness of drug testing policy<br>• Students' predicted effectiveness to reduce drug use | • Perceived drug problem found as robust predictor of perceived policy fairness.<br>• Most students perceived that drug testing would be effective to reduce drug use. |
| Evans-Whipp, Bond, Toumbourou, and Catalano (2007) | Cross-sectional survey | International Youth Development Study data 2003:<br>• 104 schools, Washington, USA<br>• 101 schools, Victoria, Australia | Washington:<br>1,934 students<br>1,886 parents<br>Victoria:<br>1,942 students<br>1,858 parents | Drug testing program | • Students' reported drug use<br>• Parents' and students' awareness of policy | • Drug testing policy associated with decreased student drug use<br>• The message of harm reduction associated with reduced drug use<br>• Parents and students aware of school policy orientation |
| Goldberg, Elliot, MacKinnon, Moe, Kuehl, Yoon, Taylor, and Williams (2007) | 2-year prospective randomized controlled study of a single cohort | 14 school districts, Oregon, USA | • 653 students in five high schools with drug testing<br>• 743 students in six control schools | Random drug and alcohol testing in high school athletes | • Students' past-year reported drug use<br>• Students' past-month reported drug use | Reduced students' past-year drug use in two of four follow-ups |
| Barrington (2008) | Quasi-experimental mixed-methods sequential explanatory design | Two rural, low-income public secondary school districts, USA | 1,048 high school students from 6th to 12th grade, and four school administrators | Voluntary, randomized, student drug testing program | Drug testing efficacy | *Qualitative findings*:<br>• Students with intensive drug abuse service needs identified<br>• Enhance school bonding and connectedness |
| Ringwalt, Vincus, Ennett, Hanley, Bowling, Yacoubian, and Rohrbach (2009) | Cross-sectional survey in spring 2005 | School districts from a national random sample, USA | 1,612 drug prevention coordinators from 1,922 school districts | Suspicion-less random drug testing implemented in 205 school districts | School districts' responses to students' first positive drug test | *Appropriate responses*:<br>• Refer students and parents to meet with school personnel and counselor<br>• Require students to receive drug education and treatment |

**TABLE 3**
**A Summary of Findings that Oppose School Drug Testing**

| Study | Study Design | Setting | Sample | Intervention | Outcomes assessed | Findings |
|---|---|---|---|---|---|---|
| Coombs and Ryan (1990) | Cross-sectional survey and in-depth interview | 21 intercollegiate teams, UCLA, USA | 624 intercollegiate athletes | Drug testing program | • Identification of students' drug use<br>• Students' reported continued drug use | • Elevated level of drug use reported in some students<br>• Ways to avoid detection of drugs reported in some students |
| Coombs and Coombs (1991) | Cross-sectional survey and in-depth interview | 21 intercollegiate teams, UCLA, USA | 500 student athletes | Mandatory drug testing for intercollegiate athletes | • Students' morale and psychological well-being<br>• Improvement in drug testing experience | *Negative feelings reported*:<br>• Embarrassed and humiliated<br>*Suggested improvements*:<br>• Orientation and education<br>• Comfortable testing setting<br>• Reasonable objectives<br>• Rigorous testing standards |
| McKinney (2002) | Cross-sectional survey | 83 high schools, Indiana, USA | 83 high school principals | Mandatory, drug testing in 1999–2000 | Students' reported illicit drug use in 2000–2001 | Students' reported drug use unchanged |
| McKinney (2003) | Cross-sectional survey McKinney's (2002) follow-up | 59 high schools, Indiana, USA | 59 high school principals | Mandatory, random drug testing program | Students' reported drug use when drug testing reimplemented | Reported drug use unchanged in some students |
| Goldberg, Elliot, MacKinnon, Moe, Kuehl, Nohre, and Lockwood (2003) | Longitudinal survey 1999–2000 | Two high schools, Oregon, USA | Athletes vs. nonathletes:<br>T1: 276 vs. 507<br>T2: 159 vs. 338 | Mandatory drug testing program for student athletes | • New drug use<br>• Students' attitude and beliefs on drug testing<br>• Students' attitude toward school | • No difference in new drug use between control and intervention schools<br>• Beliefs in reduced risk of drugs increased<br>• Negative attitude toward school increased |
| Yamaguchi, Johnston, and O'Malley (2003). | Cross-sectional national survey from 1998 to 2001 | Monitoring the Future study<br>Youth, Education, and Society study<br>USA | Monitoring the Future study:<br>76,000 students from 8th, 10th, & 12th grades | School drug testing program | • Prevalence of students' reported illicit drug use<br>• Rate of students' reported marijuana use | *Drug testing not associated*:<br>• Prevalence of students' reported illicit drug use<br>• Rate of drug use in experienced marijuana users |
| Evans, Reader, Liss, Wiens, and Roy (2006) | Cross-sectional survey conducted before drug testing implementation | Two rural high schools, North Florida, USA | 1,011 students from 9th to 11th grade | Random suspicion-less drug testing program | Students' perceived fairness of drug testing policy | *Better acceptance of drug testing should address*:<br>• Students' perceptions of peer drug use<br>• Drug testing accuracy<br>• Equitability of drug testing consequences |
| Goldberg, Elliot, MacKinnon, Moe, Kuehl, Yoon, Taylor, and Williams (2007) | 2-year prospective randomized controlled study of a single cohort | 14 school districts, Oregon, USA | 653 students in five high schools with drug testing<br>743 students in six control schools | Random drug and alcohol testing in high school athletes | Students' past-month reported drug use | • No deterrent effects for past-month drug use in any of the four follow-ups<br>• Increased risk factors for future drug use |
| Barrington (2008) | Quasi-experimental, mixed-methods sequential explanatory design | Two rural, low-income public secondary school districts, USA | 1,048 high school students from 6th to 12th grade, and four school administrators | Voluntary, randomized, student drug testing program | Students' reported drug use | *Quantitative finding*:<br>• No significant impact on students' reported drug use |
| What Works Clearinghouse (2008, May) | Review of Goldberg et al.'s (2007) study | 14 school districts, Oregon, USA | 653 students in five schools with drug testing<br>743 students in six control schools | Random drug and alcohol testing in high school athletes | • Sample attrition rate<br>• Demographic data of sample | *Inconclusive results of Goldberg et al.'s (2007) study*:<br>• High attrition rate<br>• Biased sampling |
| Ringwalt, Vincus, Ennett, Hanley, Bowling, Yacoubian, and Rohrbach (2009) | Cross-sectional survey in spring 2005 | School districts from a national random sample, USA | 1,612 drug prevention coordinators from 1,922 school districts | Random drug testing in 205 school districts | School districts' responses to students' first positive drug test | *Less appropriate responses*:<br>• Inform law enforcement<br>• Suspension from athletic team or school |

experimental schools may step up antidrug measures in schools, which would eventually exaggerate the treatment effect of school drug testing. Fourth, political and community responses to a drug testing scheme may influence student attitudes before, during, and after the implementation process. Fifth, the choice of outcome measures and honest disclosure of substance abuse behavior will definitely affect the evaluation outcomes. Sixth, as adolescent substance abuse may have a low base rate in places like Hong Kong, it may be difficult to detect real differences between the experimental group and control group unless very large sample sizes and sensitive measures are used. Finally, researchers have to consider carefully whether "blinding" can be feasibly and meaningfully carried out in related experimental studies.

The second line of research is qualitative evaluation studies. Besides those qualitative findings reported in academic journals (Tables 2 and 3), there are numerous qualitative accounts of the value and problems of school drug testing. For example, while a high school principal pointed out that "the committee worked very hard to provide a tool which would have a positive effect on our students. The extremely low number of positive tests indicates the program is worth the cost"[10, p. 1], Knight and Levy[11] warned that the view that drug testing in schools can prevent adolescent substance abuse has to be interpreted with caution because their efficacy has not yet been proven and drug tests are associated with significant technical concerns.

**TABLE 4**
**A Summary of the Quality of Studies Under Review**

| Study | Study Design | Comments on the Study |
|---|---|---|
| Coombs and Ryan (1990) | Cross-sectional survey and in-depth interview | • Cause-effect inference could not be drawn from the findings<br>• Details of group comparison not clear<br>• Samples not randomly drawn<br>• Psychometric properties of the assessment tools unclear<br>• Qualitative orientation of the study not clear<br>• Unclear about how ideological preoccupation and biases were dealt with<br>• Qualitative analysis procedures unclear<br>• Limitations of the study not properly addressed |
| Coombs and Coombs (1991) | Cross-sectional survey and in-depth interview | • Cause-effect inference could not be drawn from the findings<br>• Psychometric properties of the assessment tools unclear<br>• Samples not randomly drawn<br>• Descriptive statistics the main form of analyses<br>• Qualitative orientation of the study not clear<br>• Qualitative analysis procedures unclear<br>• Unclear about how ideological preoccupation and biases were dealt with<br>• Limitations of the study not properly addressed |
| DuPont, Campbell, and Mazza (2002) | Cross-sectional survey (quantitative and qualitative data collected) | • Cause-effect inference could not be drawn from the findings<br>• Psychometric properties of the assessment tools unclear<br>• Samples not randomly drawn<br>• Descriptive statistics the main form of analyses<br>• Qualitative orientation of the study not clear<br>• Unclear about how ideological preoccupation and biases were dealt with<br>• Qualitative data analysis procedures not clear<br>• Limitations of the study not properly addressed |
| McKinney (2002) | Cross-sectional survey | • Cause-effect inference could not be drawn from the findings<br>• Psychometric properties of the assessment tools unclear<br>• Samples not randomly drawn<br>• Descriptive statistics the main form of analyses<br>• Research report very brief<br>• Findings on the impact of random student drug-testing programs not robust – many confounding factors would affect the results<br>• Limitations of the study not properly addressed |
| McKinney (2003) | Cross-sectional survey<br>McKinney's (2002) follow-up | • Cause-effect inference could not be drawn from the findings<br>• Psychometric properties of the assessment tools unclear<br>• Samples not randomly drawn<br>• Procedures for data collection not clear<br>• Descriptive statistics the main form of analyses<br>• Research report very brief<br>• Effect of reimplementation of drug testing programs not properly evaluated<br>• Alternative explanations not properly evaluated<br>• Limitations of the study not properly addressed |
| McKinney (2005) | Cross-sectional survey<br>McKinney's (2003) follow-up | • Cause-effect inference could not be drawn from the findings<br>• Psychometric properties of the assessment tools unclear<br>• Samples not randomly drawn<br>• Hypotheses of the study not clearly stated<br>• Procedures not systematically presented<br>• Descriptive statistics the main form of analyses<br>• No details about inferential statistics used<br>• Research report very brief<br>• Alternative explanations of the findings not discussed<br>• Limitations of the study not properly addressed<br>• The conclusion that drug testing policies are effective not adequately supported by the evidence presented |
| Mason (2003) | Cross-sectional survey | • Cause-effect inference could not be drawn from the findings<br>• Psychometric properties of the assessment tools unclear<br>• Samples not randomly drawn<br>• Sample errors associated with the percentage data not properly addressed |

*Table 4 continues*

**TABLE 4 (continued)**

| Study | Study Design | Comments on the Study |
|---|---|---|
| Goldberg, Elliot, MacKinnon, Moe, Kuehl, Nohre, and Lockwood (2003) | Longitudinal survey 1999–2000 | • Longitudinal design commendable<br>• Inclusion of a comparison school methodologically superior<br>• Cause-effect inference could not be drawn from the findings<br>• Some of the measures had low internal consistency<br>• Validity of the outcome measures in both groups not clear<br>• Samples not randomly drawn<br>• Only one experimental school and one control school involved<br>• Only mandatory drug testing among the athletes focused upon<br>• Only quantitative data collected<br>• Subject attrition effect not fully explored |
| Evans, Reader, Liss, Wiens, and Roy (2006) | Cross-sectional survey conducted before drug testing implementation | • Cause-effect inference could not be drawn from the findings<br>• Reliability of the 10-item measure not particularly high<br>• Validity of the 10-item measure not clear<br>• Samples not randomly drawn<br>• Predictors of fairness attitude and policy effectiveness belief examined<br>• Limitations of the study discussed<br>• Only quantitative data collected |
| Evans-Whipp, Bond, Toumbourou, and Catalano (2007) | Cross-sectional survey | • Cause-effect inference could not be drawn from the findings<br>• Students, parents, and administrators recruited<br>• Large sample size in different samples<br>• Random and representative samples drawn<br>• Psychometric properties of the instruments not clear<br>• Both descriptive and inferential statistical analyses conducted<br>• Limitations of the study discussed<br>• Only quantitative data collected |
| Goldberg, Elliot, MacKinnon, Moe, Kuehl, Yoon, Taylor, and Williams (2007) | Two-year prospective randomized controlled study of a single cohort | • Two-year prospective randomized controlled study<br>• Reliability of measures acceptable<br>• Validity of measures not clear<br>• Limitations of the study discussed<br>• Linear mixed models not employed<br>• Only quantitative data collected |
| Barrington (2008) | Quasi-experimental mixed-methods sequential explanatory design | • A mixed-method design adopted<br>• Validated measures used<br>• Samples not randomly drawn<br>• Qualitative orientation of the study not clear<br>• Unclear about how ideological preoccupation and biases were dealt with<br>• Limitations of the study addressed |
| Ringwalt, Vincus, Ennett, Hanley, Bowling, Yacoubian, and Rohrbach (2009) | Cross-sectional survey in spring 2005 | • Cause-effect inference could not be drawn from the findings<br>• Random samples selected<br>• Data collection procedures well designed<br>• Descriptive statistics the main form of analyses<br>• Design and statistical analyses strong<br>• Limitations of the study addressed<br>• Only quantitative data collected |
| Yamaguchi, Johnston, and O'Malley (2003). | Cross-sectional national survey from 1998 to 2001 | • Cause-effect inference could not be drawn from the findings<br>• Large sample size in different samples<br>• Hierarchical linear models examined<br>• Psychometric properties of the instruments not clear<br>• Background confounding factors not properly examined<br>• Limitations of the study discussed<br>• Only quantitative data collected |
| What Works Clearinghouse (2008, May) | Review of Goldberg et al.'s (2007) study | • Sample attrition problem leading to bias<br>• Noncompletion of questionnaires leading to bias<br>• Initial differences between the experimental and control groups might create confounding effect<br>• The conclusion of the study does not conform to What Works Clearinghouse standards |

When researchers conduct qualitative evaluations of school drug testing, it is important to pay particular attention to the rigor of the studies. Shek et al.[12] pointed out that there are 12 principles that should be upheld in qualitative evaluation studies: (1) statement of the philosophical base of the study; (2) justification for the number and nature of the participants of the study; (3) detailed description of data collection procedures; (4) discussion of biases in the study; (5) description of steps taken to guard against biases or arguments that biases should and/or could not be eliminated; (6) pay attention to reliability issues; (7) considering triangulation strategies; (8) use of peer checking and member checking; (9) use of audit trails; (10) examination of alternative explanations; (11) accounting for negative evidence; and (12) examination of limitations of the study. Obviously, methodological rigor of future qualitative evaluation studies in this field can be strengthened by upholding these principles.

Adopting a balanced perspective, school drug testing schemes may not be a panacea for adolescent substance abuse. In the long run, effort should be made to integrate school drug testing with other preventive measures, such as preventive drug education and positive youth development[13,14,15,16,17], to help young people to stay away from drugs. Fundamentally, it is important to take an evidence-based approach to evaluate the strategies to tackle adolescent substance abuse, including a school drug testing scheme.

### ACKNOWLEDGMENTS

The preparation for this paper was financially supported by The Hong Kong Jockey Club Charities Trust.

## APPENDIX 1

### Studies and Articles Under Review

| Review Authors | Year | Publication Details |
|---|---|---|
| Ringwalt, C., Vincus, A.A., Ennett, S.T., Hanley, S., Bowling, J.M., Yacoubian, G.S., and Rohrbach, L.A. | 2009 | Responses to positive results from suspicion-less random drug tests in US public school districts. *J. Sch. Health* **79(4),** 177–183. |
| Barrington, K. | 2008 | Voluntary, randomized, student drug-testing: impact in a rural, low-income, community. *J. Alcohol Drug Educ.* **52(1),** 47–66. |
| What Works Clearinghouse | 2008, May | WWC quick review of the article "Outcomes of a Prospective Trial of Student-Athlete Drug Testing: The Student Athlete Testing Using Random Notification (SATURN) Study". Institute of Education Science: U.S. Department of Education. Retrieved from http://www.ies.ed.gov/ncee/wwc/publications/quickreviews/Saturn/index.asp |
| Evans-Whipp, T.J., Bond, L., Toumbourou, J.W., and Catalano, R.F. | 2007 | School, parent, and student perspectives of school drug policies. *J. Sch. Health* **77(3),** 138–146, 153–154. |
| Goldberg, L., Elliot, D. L., MacKinnon, D.P., Moe, E., Kuehl, K.S., Nohre, L., and Lockwood, C.M. | 2007 | Outcomes of a prospective trial of student-athlete drug testing: the Student Athlete Testing Using Random Notification (SATURN) study. *J. Adolesc. Health* **41,** 421–429. |
| Evans, G.D., Reader, S., Liss, H.J., Wiens, B.A., and Roy, A. | 2006 | Implementation of an aggressive random drug-testing policy in a rural school district: student attitudes regarding program fairness and effectiveness. *J. Sch. Health* **76(9),** 452–458. |
| McKinney, J.R. | 2005 | Effectiveness of Student Random Drug-Testing Programs. The Student Drug-Testing Coalition. U.S. |
| Goldberg, L., Elliot, D.L., MacKinnon, D.P., Moe, E.L., Kuehl, K.S., Yoon, M., Taylor, A., and Williams, J. | 2003 | Drug testing athletes to prevent substance abuse: background and pilot study results of the SATURN study. *J. Adolesc. Health* **32,** 16–25. |
| Mason, K. | 2003 | Drug Testing in Schools: Attitudes of High School Students [Ph.D. thesis]. University of New Orleans. (Cited in McKeganey, N. [2005] *Random Drug Testing of School Children: A Shot in the Arm or a Shot in the Foot for Drug Prevention*. Joseph Rowntree Foundation, York. pp. 7–8). |
| McKinney, J.R. | 2003 | The Effectiveness of Random Drug Testing Programs: A Statewide Follow-Up Study. The Student Drug-Testing Coalition. U.S. |
| Yamaguchi, R., Johnston, L.D., and O'Malley, P.M. | 2003 | Relationship between student illicit drug use and school drug-testing policies. *J. Sch. Health* **73(4),** 159–164. |
| DuPont, R.L., Campbell, T.G., and Mazza, J.J. | 2002 | Report of a Preliminary Study: Elements of a Successful School-Based Student Drug Testing Program. The Institute for Behavior and Health. U.S. |
| McKinney, J.R. | 2002 | The Effectiveness and Legality of Random Drug Testing Policies. The Student Drug-Testing Coalition. U.S. |
| Coombs, R.H. and Coombs, C.J. | 1991 | The impact of drug testing on the morale and well-being of mandatory participants. *Int. J. Addict.* **26(9)**, 981–992. |
| Coombs, R.H. and Ryan, F.J. | 1990 | Drug testing effectiveness in identifying and preventing drug use. *Am. J. Drug Alcohol Abuse* **16**, 173–184. |
